# Regulation of Neurotransmitters by the Gut Microbiota and Effects on Cognition in Neurological Disorders

**DOI:** 10.3390/nu13062099

**Published:** 2021-06-19

**Authors:** Yijing Chen, Jinying Xu, Yu Chen

**Affiliations:** 1 Chinese Academy of Sciences Key Laboratory of Brain Connectome and Manipulation, Shenzhen Key Laboratory of Translational Research for Brain Diseases, The Brain Cognition and Brain Disease Institute, Shenzhen Institute of Advanced Technology, Chinese Academy of Sciences, Shenzhen–Hong Kong Institute of Brain Science-Shenzhen Fundamental Research Institutions, Shenzhen 518055, China; yj.chen@siat.ac.cn (Y.C.); jy.xu@siat.ac.cn (J.X.); 2 Shenzhen College of Advanced Technology, University of Chinese Academy of Sciences, Beijing 100049, China; 3 Guangdong Provincial Key Laboratory of Brain Science, Disease and Drug Development, HKUST Shenzhen Research Institute, Shenzhen–Hong Kong Institute of Brain Science, Shenzhen Fundamental Research Institutions, Shenzhen 518057, China

**Keywords:** gut microbiota, neurotransmitters, cognition, neurodegeneration, Alzheimer’s disease

## Abstract

Emerging evidence indicates that gut microbiota is important in the regulation of brain activity and cognitive functions. Microbes mediate communication among the metabolic, peripheral immune, and central nervous systems via the microbiota–gut–brain axis. However, it is not well understood how the gut microbiome and neurons in the brain mutually interact or how these interactions affect normal brain functioning and cognition. We summarize the mechanisms whereby the gut microbiota regulate the production, transportation, and functioning of neurotransmitters. We also discuss how microbiome dysbiosis affects cognitive function, especially in neurodegenerative diseases such as Alzheimer’s disease and Parkinson’s disease.

## 1. Introduction

The intestinal tract is the largest microecosystem in the human body. There are approximately 10^14^ bacteria from more than 2000 known species living in the human intestinal tract, which collectively contain more than 100 times the genomic DNA of humans [1]. In a healthy state, the gut microbiota is in a very delicate balance. Changes due to internal or external factors that interrupt this microecological balance can lead to disorders or diseases [2]. Accordingly, gut microbiota imbalances occur in various neurological disorders including Alzheimer’s disease (AD), Parkinson’s disease (PD), autism spectrum disorder, epilepsy, and major depressive disorder [2,3,4,5,6]. Patients with AD exhibit an imbalance of the gut microbiota that manifests as decreased fecal microbial diversity, lower abundance of some beneficial bacterial taxa (e.g., *Eubacterium rectale*, *Bifidobacterium*, *Dialister*), and higher abundance of potentially pathogenic microbes (e.g., *Escherichia/Shigella*, *Bacteroides*, *Ruminococcus*) [7,8,9]. Nevertheless, it is still unclear how the gut microbiota participate in the pathogenesis of neurological disorders.

The two-way communication between the gut microbiome and the brain—termed the “gut–brain axis”—is involved in neuronal development, brain function, cognitive regulation, and aging [10]. The gut–brain axis, which includes the central nervous, endocrine, and immune systems, is an information exchange network that connects the gut and brain. It can transmit information bidirectionally: “top–down” from the brain to the gut and “bottom–up” from the gut to the brain. In addition to the classical hypothalamic–pituitary–adrenal axis and endocrine pathways (i.e., intestinal peptides and hormones), there is accumulating evidence that the metabolites (e.g., short-chain fatty acids, neurotransmitters, and their precursors) produced by bacteria affect the levels of related metabolites in the brain via the blood circulation, thus regulating brain functions and cognition [10,11,12]. In addition, the gut microbiota can act on the local nervous system (e.g., enteric nerves, vagus nerve) to quickly transmit signals to the brain [13,14]. Moreover, lipopolysaccharide and other endotoxins produced by bacteria can activate the peripheral immune system (e.g., immune cell activation, cytokine release, etc.) to promote the infiltration of peripheral immune cells into the brain, thus triggering central nervous system inflammation [12].

The recent development in meta-analysis in combination with multiple technologies such as single-cell sequencing, 16s rDNA sequencing, mass spectrometry-based metabolomics including liquid chromatography-mass spectrometry, gas chromatography-mass spectrometry, and imaging mass spectrometry, has revealed potential links between gut microbiota and the metabolism and cognition of the host [15,16,17,18]. In addition, the advance of molecular technologies including in vitro bacterial culture system [19], in vitro primary intestinal cell culture system [13], and in vivo studies in animal models [20,21] offers great opportunities to study the effects of specific bacterial taxa and their metabolites on the pathogenesis of diseases, and to develop new therapeutic strategies including probiotics and prebiotics to treat neurological disorders.

**Figure 1:**
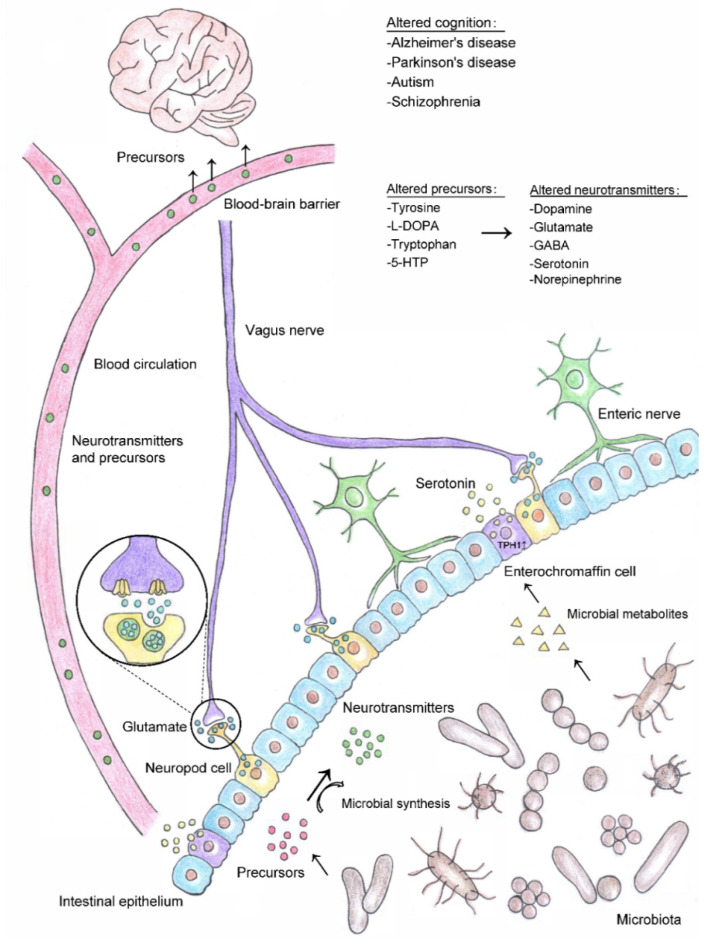
Gut microbial-mediated neurotransmitter synthesis and its impacts on cognition. Gut microbiota can either produce neurotransmitter precursors, catalyze the synthesis of neurotransmitters through dietary metabolism, or in combination. Some bacterial taxa may signal through their metabolites to promote the synthesis and release of neurotransmitters by enteroendocrine cells (e.g., metabolites produced by spore-forming bacteria serve as signaling molecules to regulate the biosynthesis of serotonin by increasing the expression of its rate-limiting gene *TPH1* in enterochromaffin cells). Neurotransmitters synthesized by bacteria and enteroendocrine cells can enter the blood circulation and be transported to other parts of the body. Some neurotransmitter precursors can cross the blood–brain barrier and participate in the synthesis cycle of neurotransmitters in the brain. In addition, neuropod cells located in the intestinal epithelium synthesize and release neurotransmitters such as glutamate, which can transmit sensory signals to the brain within milliseconds through vagus nerve. Gut microbiota-modulated changes in neurotransmitter/precursor synthesis may lead to alterations in brain function and influence cognition in neurological diseases such as Alzheimer’s disease, Parkinson’s disease, autism, and schizophrenia. Abbreviations: 5-HTP, 5-hydroxytryptophan; l-DOPA, l-3,4-dihydroxy-phenylalanine; GABA, gamma-aminobutyric acid.

This review focuses on the regulatory mechanisms of the intestinal microbiome and its metabolites (mainly neurotransmitters and their precursors) on cognitive functions and the pathogeneses of neurodegenerative diseases such as AD and PD (Figure 1).

## 2. Microbiota and Neurotransmitters

During the long process of evolution, the microbes that inhabit the gut have established symbiotic relationships with their hosts. The gut microbiota digests the host’s dietary components to meet its own nutritional needs while simultaneously providing energy and nutrients for the host. However, the gut microbiota also produces some neuroactive metabolites such as neurotransmitters or their precursors, which can affect the concentrations of either related neurotransmitters, their precursors, or both, in the brain [22,23,24,25]. This suggests that the neurotransmitter synthesis pathway in the intestine might directly or indirectly affect the neuronal activity and cognitive functions of the brain [12,26,27].

The functional activities of the brain depend on the signal transmission between different types of neurons and glial cells, which mainly depends on neurotransmitters. There are excitatory neurotransmitters such as glutamate, acetylcholine, and dopamine as well as inhibitory neurotransmitters such as γ-aminobutyric acid (GABA), glycine, and serotonin. Neurotransmitters are actively involved in various brain functions including movement, emotion, learning, and memory [26,28,29,30]. Imbalances of these neurotransmitters can lead to neurological and psychological disorders such as AD, PD, autism spectrum disorder, anxiety disorders, and depressive disorders. Therefore, investigating the dysregulation of neurotransmitter synthesis in the central nervous system and in the peripheral organs may yield novel insights into the molecular bases of these diseases and disorders.

Different types of neurons or glial cells contain specific enzymes that can catalyze the synthesis of neurotransmitters in the brain. Interestingly, certain bacterial taxa in the gut also produce enzymes that can facilitate the synthesis of neurotransmitters or their precursors. These neurotransmitter precursors can pass through the blood–brain barrier to enter the brain where they participate in the synthetic cycles of various neurotransmitters [22]. In addition, some gut bacteria can signal via their metabolites to regulate the synthesis and release of neurotransmitters by intestinal enteroendocrine cells, which can act locally on the enteral nervous system or transmit fast signals to the brain via the vagus nerve [13,30,31]. However, it remains unclear how abnormal neurotransmitter levels in the brain are associated with deficits in the synthesis of neurotransmitters or their precursors in the intestine.

## 3. Influence of Gut Microbe-Regulated Neurotransmitter Synthesis on Cognition

Neurotransmitters are chemicals that can be transported between neurons via synapses to carry messages to control behaviors such as motility, emotion, memory, etc. These chemical messengers can have excitatory or inhibitory effects on neurons. Some neurotransmitters contribute to the mutual interactions between the gut microbiota and host, and the synthesis of neurotransmitters is influenced by the microbial control of neurotransmitter precursors. Accordingly, this section focuses on the mechanisms whereby the gut microbiota regulates the synthesis of neurotransmitters and their precursors as well as the microbe-mediated synthesis and secretion of neurotransmitters by the intestinal enteroendocrine cells. This section also discusses how gut-derived neurotransmitters act on the brain through the blood circulation, local stimulation of the enteric nervous system, and rapid signal transduction via the vagus nerve (Table 1).

### 3.1. Synthesis and Functions of Neurotransmitters Modulated by the Gut Microbiota

Besides short-chain fatty acids and bile acids, recent studies show that the metabolites produced by the gut microbiota also include some neurotransmitters such as glutamate, GABA, serotonin, and dopamine [19,29,32]. Moreover, some bacteria encode genes for specific enzymes that can catalyze the conversion of substrates into corresponding neurotransmitters or precursors [31,32,33]. Meanwhile, some bacterial metabolites can act as signaling molecules to induce the synthesis and release of neurotransmitters by enteroendocrine cells [31]. As neurotransmitters such as glutamate, GABA, dopamine, and serotonin do not penetrate the blood–brain barrier, they must be synthesized in the brain from local pools of neurotransmitter precursors. Most of these precursors are amino acids (e.g., tyrosine and tryptophan) derived from the diet, which enter the blood, are transported across the blood–brain barrier, and are taken up by corresponding neurotransmitter-producing cells. The precursors are then converted into functional neurotransmitters including dopamine, norepinephrine, and serotonin through a few intermediate steps with the help of various host enzymes. Accordingly, the dietary origins of these precursors enable the intestinal microbiome to influence host behavior by regulating the metabolism of the neurotransmitter precursors.

#### 3.1.1. Glutamate

Glutamate, the most abundant excitatory neurotransmitter in the brain, is responsible for sending signals between nerve cells [34]. As glutamate cannot pass through the blood–brain barrier, its synthesis in the brain depends on the cooperation between neurons and astrocytes, which utilize the intermediate metabolites of the tricarboxylic acid cycle as precursors [35]. However, elsewhere in the body—for example, the intestinal tract—cells other than neurons can also produce glutamate. A subpopulation of enteroendocrine cells in the intestine was recently reported to synthesize glutamate and use it to transfer rapid signals to the brain via the vagus nerve [13]. The enteroendocrine cells that synapse with the vagal neurons are called “neuropod cells,” which express VGLUT1 (vesicular glutamate transporter 1) transcript and release glutamate to transduce sensory stimuli from sugars in the gut to the brain within milliseconds. Interestingly, some metabolites of the intestinal microbiota can cross the blood–brain barrier, accumulate in specific regions of the central nervous system, and participate in the biosynthesis of neurotransmitters. Notably, gavage administration of ^13^C-labeled inulin in mice shows that ^13^C acetate, a metabolite produced by carbohydrate fermentation in the gut, can cross the blood–brain barrier, accumulate mostly in the hypothalamus, and participate in the neuronal–glial cycle of glutamate–glutamine metabolic coupling in the hypothalamus [22].

**Table 1:** Gut microbiota-regulated neurotransmitter synthesis and functions in the gut–brain axis. A list of specific neurotransmitters and their precursors, synthesis in particular bacterial taxa or in intestinal cells, and the putative functions of these neurotransmitters in the gut-brain axis. Abbreviations: 5-HTP, 5-hydroxytryptophan; GABA, gamma-aminobutyric acid; l-DOPA, l-3,4-dihydroxy-phenylalanine; PD, Parkinson’s disease.

Neurotransmitters	Precursors	Gut Microbiota	Intestinal Cells	Putative Functions in the Gut–Brain Axis
Glutamate	Acetate	*Lactobacillus plantarum* [36] *Bacteroides vulgatus* [36] *Campylobacter jejuni* [36]	Enteroendocrine cells [13]	Transfer intestinal sensory signals to the brain through the vagus nerve [13]
GABA	Acetate	*Bifidobacterium* [19] *Bacteroides fragilis* [19] *Parabacteroides* [19] *Eubacterium* [19]	Myenteric neurons [37] Mucosal endocrine-like cells [38]	Modulate synaptic transmission in the enteric nervous system [37] Modulate intestinal motility and inflammation [38]
Acetylcholine	Choline	*Lactobacillus plantarum* [39] *Bacillus* *acetylcholini* [40] *Bacillus subtilis* [41], *Escherichia coli* [41] *Staphylococcus aureus* [41]	Myenteric neurons [42,43,44]	Produced by 33% myenteric neurons in human colon [44] Regulate intestinal motility and secretion [42] and enteric neurotransmission [43]
Dopamine	Tyrosine l-DOPA	*Staphylococcus* [32]		Affect gastric secretion, motility, and mucosal blood flow [45] Affect gastric tone and motility through nigro-vagal pathway in a Parkinson’s disease (PD) rat model [46]
Serotonin	5-HTP Tryptophan	*Staphylococcus* [32] *Clostridial species* [31]	Enterochromaffin cells [47]	Promote intestinal motility [48]
Norepinephrine	Tyrosine			Modulate energy intake and thermal homeostasis [49]
Tyramine	Tyrosine	*Staphylococcus* [32] *Providencia* [50]		Precursor of octopamine [50]
Phenylethylamine	Phenylalanine	*Staphylococcus* [32]		
Tryptamine	Tryptophan	*Staphylococcus* [32] *Ruminococcus gnavus* [33] *Clostridium sporogenes* [33]		Induce serotonin secretion by enterochromaffin cells [51] Promote gastrointestinal transit and colonic secretion [52]

#### 3.1.2. GABA

GABA is an inhibitory neurotransmitter that participates in various metabolic and physiological activities. In the brain, GABA is synthesized by GABAergic neurons, which convert glutamate into GABA via the enzyme glutamic acid decarboxylase produced exclusively in these neurons [53]. The fact that GABA can be produced in the gut by certain bacterial taxa was discovered accidently. A recent study involving a co-culture method to screen for critical growth factors required for bacterial survival isolated gram-positive bacteria belonging to Ruminococcaceae (designated “KLE1738”) from the human gut microbiota that requires *Bacteroides fragilis* to grow [19]. It was subsequently discovered that GABA is the main growth factor produced by *B.* *fragilis*. Moreover, this co-culture system revealed that *Parabacteroides*, *Eubacterium*, and *Bifidobacterium* also synthesize GABA. Subsequent transcriptomic analysis of human fecal samples corroborates that GABA is synthesized by these microbes [19]. However, GABA does not cross the blood–brain barrier either. Therefore, gut microbe-derived GABA might act locally on the enteric nervous system or the vagus nerve as it cannot enter the brain. However, similar to the glutamate synthesis pathway in the central nervous system, metabolites of the colonic fermentation of carbohydrates by the microbiota, such as acetate, can cross the blood–brain barrier and be incorporated into the GABA metabolic cycle, preferentially in the hypothalamus [22].

#### 3.1.3. Acetylcholine

Acetylcholine is a common cholinergic neurotransmitter in vertebrates and insects that functions as a local mediator in the central and peripheral nervous systems by transducing excitatory signals between neurons [54]. Its dysregulation is closely associated with neurodegenerative diseases such as AD [55,56]. Acetylcholine was first discovered in a study of ergot on wheat rye in the early 1900s, although it was only later noticed that *Bacillus acetylcholini* rather than ergot actually produced this neurochemical [40]. Since then, acetylcholine has been found to be produced by multiple bacteria including *Lactobacillus plantarum*, *Bacillus subtilis*, *Escherichia coli*, and *Staphylococcus aureus* [39,41]. Notably, *B. subtilis* contains larger quantities of acetylcholine than *E. coli* or *S. aureus* [41]. As acetylcholine cannot cross the blood–brain barrier, neurons in the central nervous system synthesize acetylcholine from choline and acetyl coenzyme A catalyzed by choline acetyltransferase [57]. Peripherally derived choline can be transported to the brain via the carriers located on capillary endothelial cells [58].

#### 3.1.4. Dopamine

Within the central nervous system, dopamine is mainly produced in the substantia nigra and ventral tegmental areas in the brain. Several neurological disorders such as schizophrenia and PD are characterized by dysregulation of the dopamine system [59,60]. Being the most abundant catecholamine neurotransmitter in the brain, dopamine is synthesized in dopaminergic neurons from tyrosine, which is abundant in diets and can be transported to the brain via the blood–brain barrier. Outside the brain, dopamine production has been detected in *Staphylococcus* in the human intestine, which can take up the precursor l-3,4-dihydroxy-phenylalanine (l-DOPA) and convert it into dopamine by staphylococcal aromatic amino acid decarboxylase (SadA) expressed by these bacteria [32]. More than 50% of dopamine in the human body is synthesized in the gut [61]. Dopamine and its receptors are widely distributed in the intestinal tract and affect gastric secretion, motility, and mucosal blood flow [62,63].

#### 3.1.5. Serotonin

In the central nervous system, serotonin is mainly synthesized by serotonergic neurons in the raphe nuclei. Abnormal expression and function of serotonin in the brain are associated with the pathogenesis of mental health disorders including depressive and anxiety disorders [64]. Of note, approximately 90% of serotonin is synthesized in peripheral parts of the human body, mainly by enterochromaffin cells in the intestinal epithelium. However, serotonin cannot cross the blood–brain barrier, but its precursor tryptophan can. In the gut, enterochromaffin cells take up tryptophan from dietary protein as substrate to synthesize serotonin, and this process is regulated by the bacterial kynurenine synthesis pathway [47,65]. Spore-forming bacteria in the gut (predominantly *Clostridia*) can promote the biosynthesis of serotonin by increasing the gene expression of its rate-limiting enzyme tryptophan hydroxylase 1 (*TPH1*) in colonic enterochromaffin cells, and certain metabolites produced by these spore-forming bacteria have been identified as the signaling molecules that trigger this action [31]. Serotonin production has also been detected in staphylococci, which use SadA to decarboxylate the precursor 5-hydroxytryptophan (5-HTP) into serotonin [32].

#### 3.1.6. Trace Amines

Trace amines (e.g., octopamine, phenylethanolamine, tyramine, tryptamine, and synephrine) are a subgroup of biogenic amines that can act as neuromodulators or neurotransmitters, although their abundance in the brain is low. Large quantities of staphylococcal strains that produce trace amines were recently discovered in the human intestine [32]. Notably, SadA-expressing staphylococci produce three types of trace amines—tryptamine, tyramine, and phenylethylamine—by decarboxylation of their corresponding aromatic amino acid substrates—tryptophan, tyrosine, and phenylalanine, respectively [32].

One example of how bacteria-derived trace amines can modulate host behaviors comes from a recent study of *C. elegans*. Commensal *Providencia* that colonize the intestine of the nematode *C. elegans* can synthesize tyramine, which is then converted into octopamine in the presence of tyramine β-hydroxylase enzyme produced by the nematode. Octopamine subsequently binds to its receptor OCTR-1 on ASH nociceptive neurons in *C. elegans* to reduce avoidance of the aversive alcohols produced by *Providencia*, thus altering the host’s food choices by biasing them towards these bacteria [50].

In addition, tryptamine, a β-arylamine neurotransmitter with low quantities in the brain, participates in various neuronal activities [66]. Tryptamine in the gastrointestinal tract can induce serotonin secretion by enterochromaffin cells, which are important for gastrointestinal motility [51]. *Clostridium sporogenes* and *Ruminococcus gnavus* from phylum Firmicutes, which are found in the human intestine, were recently identified to produce tryptamine by decarboxylating tryptophan using their own tryptophan decarboxylase [33]. As tryptophan can cross the blood–brain barrier and serves as a serotonin precursor in the brain, these findings suggest that the intestinal microbiota may influence host behavior by regulating the metabolism of tryptophan and serotonin in the brain and peripheral system.

#### 3.1.7. Norepinephrine

Norepinephrine is a catecholamine that functions as a neurotransmitter in both the central and peripheral nervous systems. It is involved in arousal, alertness, memory, and attention and also triggers the acute stress response (also called the “fight-or-flight” response) during threatening situations. In the brain, norepinephrine is mainly produced by neurons in the locus coeruleus, where the neurotransmitter precursor tyrosine is converted into dopamine and finally into norepinephrine. Interestingly, a recent study reports that alterations in the gut microbiota composition under cold temperatures can regulate norepinephrine release in the intestine and brown adipose tissue in Brandt’s voles, thereby contributing to the modulation of energy intake and thermal homeostasis during winter [49].

#### 3.1.8. Modulation of Neurotransmitter Synthesis by Gut Microbiota

The findings mentioned above collectively demonstrate an association between a healthy gut microbiome structure and balanced neurotransmitter levels in the host. Accordingly, the substantial influence of gut microbial changes on neurotransmitter synthesis has been demonstrated in both germ-free and antibiotic-treated mice. Numerous studies have revealed changes in the fecal and serum levels of neurotransmitters including GABA, serotonin, and acetylcholine as well as their precursors such as tryptophan and choline in the absence of microbial colonization (i.e., germ-free mice) [31,67,68,69,70,71]. Similarly, antibiotic administration, which leads to the acquired deprivation of gut bacteria, also alters the levels of neurotransmitters and their precursors in the gut and blood [25,72]. It is noteworthy that changes in intestinal microbial abundance also alter the expression of neurotransmitter receptors within the brain [73,74,75].

Interestingly, changes in dietary habits and environment can lead to changes in gut microbial composition, which subsequently affect neurotransmitter synthesis. To adapt to the environment when food supplies are low, some animals such as voles consume feces (i.e., coprophagia) to meet their nutritional requirements. However, voles prevented from feeding on feces exhibit decreased alpha diversity of their intestinal microbiomes, altered abundance of bacterial taxa (e.g., Firmicutes and Bacteroidetes), reduced cecal concentrations of short-chain fatty acids (e.g., acetate, propionate, and butyrate), and reduced concentrations of tyrosine hydroxylase and neurotransmitters (e.g., dopamine and serotonin) in the hippocampus and hypothalamus [76]. Other environmental factors such as a high-fat or carbohydrate-rich diet and temperature changes also significantly influence intestinal microbial composition and contribute to alterations in neurotransmitter levels in the feces, blood, and central nervous system [24,49,77].

### 3.2. Circulatory Pathway of Gut Neurotransmitter/Precursor Transport and Cognition

As mentioned above, some neurotransmitters, their precursors, or both, are synthesized in the gut either by enteroendocrine cells or the intestinal microbiota. However, it is incompletely understood how these neurotransmitters and precursors are transported to other parts of the body and eventually to the brain. Some neurotransmitter precursors synthesized in the gut that are small enough to cross the blood–brain barrier might be transported to the central nervous system via blood circulation. As its name suggests, the blood–brain barrier exists between blood vessels and the brain, and selectively prevents some substances (mostly harmful ones) from entering the brain. It comprises continuous capillary endothelium around the brain (and tight connections between its cells), a complete basement membrane, pericytes, and a glial membrane surrounded by astrocytes. The blood–brain barrier usually allows small molecules to penetrate the brain but not larger molecules such as neurotransmitters. Most neurotransmitter precursors are amino acids that can be actively transported into the brain via the carrier system of capillary endothelial cells, which can transport substances from the blood to endothelial cells. Known amino acid carriers include the neutral amino acid carrier, cationic amino acid carrier, and glutamine carrier [78]. The metabolites of spore-forming *Clostridia* were recently found to promote serotonin synthesis by colonic enterochromaffin cells. Such serotonin is released into the colon and subsequently enters the blood where it is taken up by circulating platelets and transported to other parts of the body except the brain [31]. However, the serotonin precursor tryptophan can pass through the blood–brain barrier and participate in serotonin biosynthesis in the brain. Another study revealed that ^11^C- and ^13^C-labeled acetate produced in the colon or administered in vivo through intravenous or colonic infusion entered the blood, was taken up by neuroglial cells in the hypothalamus, and is subsequently incorporated into the tricarboxylic acid, glutamate–glutamine, and GABA transcellular cycles as indicated by increased glutamate, glutamine, GABA, and lactate carbon levels [22].

These findings indicate a relationship between intestinal microbial metabolism and neurotransmitter biosynthesis in the brain. Accordingly, numerous studies demonstrate that the changes in the composition of the gut microbiota can affect the biosynthesis and metabolism of neurotransmitters or their precursors [19,23,25,49]. Such changes alter the concentrations of neurotransmitters and their precursors in the blood and subsequently affect their respective concentrations in the brain, which can disturb host brain function and cognition. A recent study found that the extracellular vesicles secreted by the gut bacteria *Paenalcaligenes hominis* are transported through the blood to the hippocampus and consequently lead to cognitive impairment in mice [79]. In addition, studies using germ-free animals have provided key information showing how the microbiome influences brain function. For instance, germ-free mice exhibit skeletal muscle atrophy accompanied by a lower serum level of choline—the precursor of acetylcholine, which plays an essential role in signal transduction between muscles and nerves at neuromuscular junctions [71]. Notably, fecal microbe transplantation from specific-pathogen-free mice to germ-free mice or treating germ-free mice with short-chain fatty acids can reverse skeletal muscle impairment [71]. These findings suggest that the intestinal microbiome might influence host behaviors by regulating neurotransmitter levels and the expression of their receptors, which are involved in synaptic function and plasticity in the brain.

In addition, the attenuation of intestinal microbiota levels by antibiotics has been used to investigate how the microbiota influences brain function. A recent study demonstrated that antibiotic administration changes the composition of the fecal microbiota in piglets and significantly reduces the levels of aromatic amino acids (i.e., tyrosine, tryptophan, and phenylalanine) in the feces, blood, and hypothalamus along with the levels of neurotransmitters derived from them, including serotonin and dopamine, in the hypothalamus [25]. In a following study, carbohydrate consumption improved the intestinal microbiome composition and increased aromatic amino acid levels in the feces, blood, and central nervous system, which consequently increased serotonin, dopamine, and brain-derived neurotropic factor levels in the hypothalamus, thus improving brain health [24].

Dietary habits and environmental changes might also contribute to the microbe-related changes in host cognition and behavior. As mentioned above, preventing coprophagia in voles alters the abundance of certain gut bacteria and decreases cognitive function, which might be associated with the changes of neurotransmitter levels in the central nervous system [76]. However, administration of the short-chain fatty acid acetate ameliorates such cognitive deficits in voles prevented from engaging in coprophagia, and this is probably because acetate restores the intestinal microbial composition and metabolic homeostasis [76]. Other dietary habits such as a high-fat diet can alter the gut microbiota and induce further neurobehavioral changes. In addition, such changes in the gut microbiota precede the inflammatory reaction from the intestine to the brain. The gut microbiota also affects neurotransmitter synthesis and brain-derived neurotropic factor levels in the brain [77]. Environmental changes such as cold acclimation can alter the composition of the gut microbiota in male Brandt’s voles, which increases the levels of neurotransmitters in the hypothalamus (e.g., norepinephrine, serotonin, and dopamine), food intake, and thermogenesis [49,77]. These findings suggest that the intestinal microbiota can affect the synthesis and blood levels of neurotransmitters to ultimately influence the levels of neurotransmitters delivered to the brain, which can in turn alter brain functions and cognition.

### 3.3. Local Regulation of Neurotransmitters and Impacts on Cognition

It was previously believed that the sensory information from the gut could only be transmitted to the brain via the release of hormones by enteroendocrine cells [30]. However, a subset of enteroendocrine cells called neuropod cells can synapse with vagal nodose neurons and release glutamate to transmit sugar stimuli to the vagus nerve [13]. Thus, the neuropod cell–vagus nerve pathway provides a more efficient gut–brain communication route than the endocrine system, thereby enabling rapid responses to the sensory circumstances derived from the local response in the gut.

Intestinal bacteria can modulate serotonin release by enterochromaffin cells, which subsequently activate the enteric nerve fibers to promote gastrointestinal motility. Microbial metabolites and other irritants are detected by the sensory receptors of enterochromaffin cells, which activates these cells and leads to serotonin secretion [31]. Meanwhile, subsets of enterochromaffin cells express the presynaptic protein synapsin and can communicate with the afferent fibers of chemosensory dorsal root neurons or vagal sensory nerves to induce a local effect of serotonin [80]. These findings suggest that the intestinal microbiome has a role in the modulation of local serotonin signaling, which impacts the central nervous system.

The extracellular vesicles secreted by *P. hominis*, whose prevalence increases in aging individuals, potentially enter the brain via the vagus nerve and cause cognitive impairment in mice [79]. Furthermore, feces from older people and older mice exhibit elevated abundance of *P. hominis* and *E. coli*. Accordingly, gavage administration of *P. hominis* and *E.* *coli*, or *P. hominis* extracellular vesicles or lipopolysaccharides to mice also causes cognitive impairment and colitis. However, abdominal vagus nerve amputation significantly inhibits cognitive deficits in such mice as well as the infiltration of extracellular vesicles into the hippocampus [79]. These findings imply that the metabolites or molecules of the gut microbiota can ascend along the vagus nerve to enter the brain. Hence, the vagus nerve not only transmits local sensory impulses mediated by the intestinal neuropod cells but also serves as a physical bridge for the microbiota–gut–brain axis. However, it remains unclear whether neurotransmitters produced in the gut can be transported to the brain via the vagal pathway. Regardless, the finding that bacteria-derived molecules or components can be transported to the brain via the vagus nerve is intriguing and opens an avenue for the development of a drug delivery system that targets the brain through the gut microbiota–vagal pathway.

Taken together, the findings mentioned above provide evidence for a microbiota–gut–vagus nerve–brain signaling pathway, suggesting that the intestinal microbiome might affect brain function and behavior via the vagal nerve system and that vagus nerve deficits might in turn affect gut–brain communication.

### 3.4. Gut Microbiota and Neurotransmitters in Neurological Disorders

#### 3.4.1. Alzheimer’s Disease and Parkinson’s Disease

Substantial recent evidence indicates that the intestinal microbial composition is altered in neurodegenerative diseases such as AD and PD [7,8,9,10], and that such alterations are accompanied by gastrointestinal disorders [81]. In the last decades, numerous studies have demonstrated an association between the dysregulation of neurotransmitters including glutamate, acetylcholine, dopamine, GABA, serotonin, and norepinephrine and the cognitive impairment in AD [36,82,83,84,85]. Interestingly, the development of in vitro bacterial culture systems has led to the discovery of several bacterial taxa that produce certain neurotransmitters, revealing a close relationship between the gut microbiota and neurotransmitter levels in the host. Increases of *Escherichia/Shigella, Bacteroides,* and *Ruminococcus* and decreases of *E. rectale, Bifidobacterium,* and *Dialister* have been detected in the feces of cognitively impaired elderly or patients with AD [7,8,9]. Notably, some of these bacterial taxa such as *E. coli*, *Bacteroides,* *Eubacterium,* and *Bifidobacterium* are involved in the production of AD-associated neurotransmitters such as acetylcholine, GABA, and glutamate [19,36,39,41], demonstrating a potential link between gut dysbiosis and neurotransmitter dysregulation in AD pathogenesis (see Table 2 for more details). In a clinical study, fecal metabolomic analysis showed a perturbed tryptophan metabolism in patients with amnestic mild cognitive impairment (aMCI) and AD [16]. The reduced serotonin precursor 5-HTP in the tryptophan pathway was positively correlated with cognitive impairment in AD. In addition, a significant correlation was observed between the disturbed gut microbiota and the fecal metabolism; for example, the abundance of *Ruminococcus* was negatively associated with the presence of indole-3-pyruvate from the tryptophan pathway. Notably, indole-3-pyruvate, which serves as a precursor for ligands of the aryl hydrocarbon receptor (AhR) signaling pathway, progressively increased from aMCI to AD patients, and was negatively correlated with cognitive function in AD. These results suggest that the gut microbiota may influence the progress of AD pathology by regulating the synthesis of neurotransmitters or the precursors of specific ligands. Furthermore, some studies show that the alterations of the intestinal microbiota in mouse models of AD are correlated with changes in the levels of fecal short-chain fatty acids such as butyric and isobutyric acid [86]. The changes in the levels of these microbial metabolites in AD mouse models are consistent with those in the brain, suggesting that intestinal microbial metabolism might affect the brain metabolic pathways in AD [86]. Importantly, studies using animal models have indicated a mutual interaction between the gut microbiota and the brain in the pathogenesis of AD. For example, injection of Aβ_1-42_ into the hippocampus of rat significantly altered the microbial composition and the intestinal transcriptome, with an increase in some proinflammatory bacteria, a reduction in some anti-inflammatory bacteria, and an activation in the immune system of the gut [87]. In turn, administration of fructooligosaccharides extracted from Morinda officinalis was able to increase the abundance of probiotic *Lactobacillus*, anti-inflammatory *Bifidobacterium*, and some other neurotransmitter-producing bacteria, accompanied by the upregulation of the brain levels of neurotransmitters including acetylcholine, dopamine, serotonin, and norepinephrine, and ameliorate cognitive impairment in AD rat model [87]. These results provide evidence for a top-down regulation of brain Aβ in the gut immune homeostasis through the gut–brain axis, and that prebiotics or probiotics may improve cognitive functions through a bottom-up pathway by targeting the gut microbiota. Consistent with this, fecal microbiota transplantation from wild-type mice into AD model mice also ameliorated cognitive impairment, accompanied by a reduction in amyloid plaques and neurofibrillary tangles [88]. More recently, there is a case report of a patient with AD who displayed rapid improvement in memory and mood after fecal microbiota transplantation from a healthy donor, providing a novel therapeutic strategy for AD treatment based on the modulation of gut microbiota [89]. These results indicate a causal association between the gut microbial composition and cognition, and the potential application of fecal microbiota transplantation in AD treatment by targeting gut microbiota homeostasis.

Meanwhile, PD is characterized by the dysregulation of the dopamine system [60]. Increase of *Akkermansia*, *Catabacter*, *Lactobacillus*, *Bifidobacterium*, Bifidobacteriaceae, Ruminococcaceae, Verrucomicrobiaceae, Christensenellaceae, and decrease of *Roseburia*, *Faecalibacterium,* Lachnospiraceae ND3007 group, Prevotellaceae, *Blautia*, *Coprococcus*, and *Lachnospira* have been observed in PD patients [17,90,91]. The metabolism of l-DOPA and dopamine is associated with the abundance of *Enterococcus faecalis* in patients with PD [92]. A recent study in a PD mouse model showed that oral administration of berberine promotes the production of the dopamine precursor l-DOPA by increasing the activity of its rate-limiting enzyme tyrosine hydroxylase in *Enterococcus faecalis* [93]. Accordingly, l-DOPA permeates the blood–brain barrier and is transformed into dopamine in the brain, thus alleviating the symptoms of PD mouse models [93]. These results suggest that oral administration of berberine can improve brain function by upregulating l-DOPA biosynthesis by the gut microbiota in PD mouse models, and might therefore be applicable as a treatment for PD in the future. In recent years, the exploration of fecal microbiota transplantation as an effective therapeutic strategy in PD treatment has obtained much attention. Several clinical trials with fecal microbiota transplantation from healthy controls to patients with PD have demonstrated significant effects on attenuating the motor symptoms, meanwhile improving non-motor symptoms such as the quality of sleep and life, and relieving anxiety and depression as well as constipation symptoms in PD [94,95,96]. It is speculated that the effects of fecal microbiota transplantation may rely on the reconstruction of gut microbiota in patients with PD. It was found that fecal microbiota transplantation on a PD mouse model gut reduces microbial dysbiosis, increases striatal neurotransmitters including dopamine and serotonin, and suppresses the activation of astrocytes and microglia in the substantia nigra region of the brain, revealing a neuroprotective effect of fecal microbiota transplantation on PD treatment [97]. Another study on a PD mouse model demonstrates a neuroprotective effect of propionate, a gut microbial metabolite, on reducing motor deficits and enhancing dopaminergic neurons in PD mice, which may exert its effects through the propionate receptor in the enteric nervous system [98].

#### 3.4.2. Autism Spectrum Disorder and Schizophrenia

Autism spectrum disorder is associated with the dysregulation of glutamate, dopamine, and serotonin in the brains of patients [99,100,101]. Increases of *Bacteroides*, *Prevotella*, *Lachnospiracea_incertae_sedis*, and *Megamonas* as well as decreases of *Clostridium XlVa*, *Eisenbergiella*, *Clostridium IV*, *Flavonifractor*, *Escherichia*/*Shigella*, *Haemophilus*, *Akkermansia*, and *Dialister* have been detected in patients with autism [102]. Glutamate metabolism is associated with the alterations of gut microbiota composition in patients with autism [36]. Recent studies have focused on establishing rodent models of autism using fecal transplantation from children with autism, which enables the investigation of the causal relationship between the gut microbiota and autism symptoms, and aids therapeutic development. For example, the transplantation of feces from children with autism induces autism-like behaviors as well as altered tryptophan and serotonin metabolism in germ-free recipient mice [103]. Another study reports that an autism rat model, transplantation with fecal samples from children with autism results in a decrease in the serum levels of GABA and norepinephrine, an increase in the serum level of serotonin, and increases in the abundance of *Lactobacillus* and *Collinsella* [104]. These results suggest that a typical gut microbial composition from patients with a certain disorder can trigger a disease symptom that is usually accompanied by alterations in neurotransmitter levels. Therefore, microbial therapy might be an efficient means to treat such disorders. Accordingly, a recent study demonstrates that *Lactobacillus reuteri* can reverse social deficits in mouse models of autism, which are abolished by subdiaphragmatic vagotomy or inactivation of oxytocin receptors in dopaminergic neurons [105]. Further investigation suggests that *L. reuteri* exerts its effect on social behavior through the oxytocinergic system, and that it reverses the impaired synaptic plasticity in dopaminergic neurons of the ventral tegmental area in an oxytocin receptor-dependent manner [105]. However, the mechanisms by which *L. reuteri* interact with the vagus nerve remains unknown. Interestingly, transplantation of fecal microbiota from wild type mice into a mouse model of autism restored the population of *Akkermansia muciniphila* and ameliorated cognitive deficits and autistic-like behaviors [106]. Deficits in the gut microbial composition and their metabolic contents such as vitamin and neurotransmitters may be involved in the development of autistic-like social behaviors in these autism mice [107]. Importantly, children with autism showed a significant improvement in autistic and gastrointestinal symptoms after treatment with fecal microbiota transplantation [108], and these benefits prolong for years as reported in a follow-up study with the same participants [109].

Meanwhile, dysregulation of the dopamine system was characterized in schizophrenia [59]. Patients with schizophrenia exhibit increased abundance of gut facultative anaerobes, which can survive in the presence or absence of oxygen, such as *Enterococcus faecium*, *Lactobacillus fermentum*, *Cronobacter sakazakii*/*turicensis*, and *Alkaliphilus oremlandii*. In addition, aberrant metabolism of short-chain fatty acids and tryptophan, and alterations of the synthesis pathways of various neurotransmitters including glutamate, GABA, dopamine, and serotonin have also been observed in these schizophrenia patients [110]. In addition, mice transplanted with fecal microbiota from patients with schizophrenia exhibit impaired learning and memory abilities that mimic those observed in the donors; such mice also exhibit lower serum levels of tryptophan and serotonin as well as higher levels of dopamine and serotonin in the prefrontal cortex and hippocampus, respectively [23]. It is interesting that the presence of a single species, *Streptococcus vestibularis*, which is associated with schizophrenia, is sufficient to induce schizophrenia-like social behaviors in recipient mice [110]. The precise mechanisms by which *S. vestibularis* induces schizophrenia-like symptoms are unclear, but these bacteria were found to be associated with the levels of brain neurotransmitters including GABA, tryptophan and glutamate in patients with schizophrenia [110]. In addition, *S. vestibularis*-treated mice show changes in the level of intestinal dopamine, GABA, and serotonin, as well as alterations in the gene expression level related to immune or inflammatory response in both the brain and the intestine, suggesting a potential role of *S. vestibularis* in the modulation of neurotransmitter synthesis and immune homeostasis during the pathogenesis of schizophrenia [110].

#### 3.4.3. Anxiety and Depressive Disorders

The deregulation of serotonin, GABA, dopamine, and glutamate levels in the central nervous system is associated with the pathogenesis of mental health disorders like anxiety [111,112,113]. Moreover, recent studies provide evidence that the gut microbiota affects anxiety symptoms. For example, germ-free mice exhibit reduced anxiety-like behaviors, which are accompanied by elevated hippocampal concentrations of serotonin and its main metabolite 5-hydroxyindoleacetic acid as well as an increased plasma concentration of its precursor tryptophan [70]. Intriguingly, these anxiolytic-like biochemical characteristics observed in germ-free mice are associated with changes in neurotransmitter receptor expression in the brain. Several studies report reduced expression of glutamate N-methyl-D-aspartate (NMDA) receptors and serotonin receptors in the cortex, hippocampus, or amygdala in germ-free mice [73,74]. Administration with probiotics such as *Lactobacillus rhamnosus* can regulate the mRNA levels of GABA_B1b_ and GABA_Aα2_ subunits in distinct regions of the brain including the cortex, the hippocampus, and the amygdala, thereby ameliorating anxiety- and depression-like behaviors in mice [75]. However, these effects are prevented in vagotomized mice, implying a crucial connection between the bacteria and the brain through the vagal nerve pathway.

Meanwhile, patients with depression exhibit dysregulation of GABA, serotonin, and dopamine levels [114,115]. GABA is largely produced by Bacteroides in the gut microbiota, and correlation analysis of 16S rRNA sequencing and fMRI data shows that the relative abundance of Bacteroides in the feces of patients with depression is negatively correlated with brain symptoms of depression [19]. Subsequent transcriptomic analysis of human fecal samples corroborates that GABA is synthesized by these microbes [19]. Moreover, patients with depression exhibit increased intestinal leakage and bacterial translocation, implying that the low-level inflammatory reaction caused by the intestinal leakage of lipopolysaccharides can lead to altered brain function and behavior [77]. Transplantation of fecal microbiota from a mouse model of depression induces depressive-like behaviors as well as altered endocannabinoid signaling in the hippocampus of germ-free recipient mice, and the complement with probiotics *Lactobacillus plantarum* is able to restore normal behaviors by increasing fatty acid precursors of endocannabinoid ligands [116]. All together, these findings indicate that the intestinal microbiota is involved in the pathogenesis of neurological disorders via their metabolites such as neurotransmitters and short chain fatty acids (SCFAs), and that prebiotics/probiotics administration may improve cognitive functions by modulating gut microbial homeostasis and metabolism.

**Table 2:** Summary of the associations between neurotransmitters and neurological disorders. A list of studies from cell culture, animal models and human cases on the modulation of neurotransmitter production by gut microbiota and its impacts on neurological disorders. Abbreviations: 5-HTP, 5-hydroxytryptophan; AD, Alzheimer’s disease; APOE, apolipoprotein E; GABA, gamma-aminobutyric acid; l-DOPA, l-3,4-dihydroxy-phenylalanine; LC-MS/MS, liquid chromatography tandem mass spectrometry; MCI, mild cognitive impairment; MPTP, methyl-4-phenyl-1,2,3,6- tetrahydropyridine; NMDA, N-methyl-D-aspartate; SadA, staphylococcal aromatic amino acid decarboxylase; SCFA, short chain fatty acid; TPH1, tryptophan hydroxylase 1.

Neurological Disorders	Neurotransmitters	Interpretations in Cell Culture/Animal Model Studies	Interpretations in Clinical Studies
Alzheimer’s disease	Glutamate, Acetylcholine, Dopamine, Serotonin, GABA, Norepinephrine	Levels of glutamate, acetylcholine, GABA, dopamine, serotonin and norepinephrine are significantly reduced in the brain of an Alzheimer’s disease (AD) rat model by LC-MS/MS analysis [117]. Increases of *Escherichia*/*Shigella*, *Desulfovibrio*, *Akkermansia*, *Blautia, Sutterella*, *Odoribacter*, and *Helicobacter* and decreases of *Prevotella* and *Butyricicoccus* have been detected in APP/PS1 mice [86,118,119,120]. Acetylcholine is produced by *Lactobacillus plantarum*, *Bacillus subtilis*, *Escherichia coli*, and *Staphylococcus aureus* [39,41]. GABA is produced by *Bacteroides* *fragilis*, *Parabacteroides*, *Eubacterium*, and *Bifidobacterium* [19]. Spore-forming bacteria in the gut (predominantly *Clostridia*) can promote the biosynthesis of serotonin by increasing the gene expression of its rate-limiting enzyme *TPH1* in colonic enterochromaffin cells [31]. Serotonin is also produced by staphylococci, which use SadA to decarboxylate the precursor 5-HTP into serotonin [32]. Glutamate metabolism is modulated by *Bacteroides vulgatus* and *Campylobacter jejuni* [36]. Significant differences in microbial-derived amino acids and SCFAs are detected between apolipoprotein E (*APOE*) genotypes by metabolomic analysis [18].	The dysregulation of glutamate, acetylcholine, dopamine, GABA, serotonin, and norepinephrine in the central nervous system is associated with cognitive impairment in AD [36,82,83,84,85].
			Increases of *Escherichia/Shigella*, *Bacteroides*, and *Ruminococcus* and decreases of *Eubacterium rectale*, *Bifidobacterium*, and *Dialister* have been detected in cognitively impaired elderly or patients with AD [7,8,9].
			Dopamine production has been detected in staphylococci in the human intestine, which can take up the precursor l-DOPA and convert it into dopamine by SadA expressed by these bacteria [32]. Significant differences in the gut microbial metabolism of tryptophan, SCFAs, and lithocholic acid were found among AD, amnestic MCI, and healthy control groups, and a metabolite of tryptophan, indole-3-pyruvic acid, is identified as a marker for AD diagnosis [16]. The abundances of Prevotellaceae and Ruminococcaceae and butyrate-producing bacteria are associated with *APOE* genotypes [18].
Parkinson’s disease	Dopamine	PD is associated with a decreased level of dopamine in the brain of PD model mice [60].	PD is characterized by the dysregulation of the dopamine system [60].
		Increases of Prevotellaceae, Erysipelotrichaceae, and Erysipelotrichales, and decreases of *Bifidobacterium*, *Lachnospiraceae*, *Clostridiales*, and Proteobacteria have been detected in an MPTP-induced PD mouse model [121]. An increase of *Lactobacillus* and decreases of *Clostridium*, *Sutterella*, and *Desulfovibrio* have been detected in a rotenone-induced PD mouse model [122].	Increases of *Akkermansia*, *Catabacter*, *Lactobacillus*, *Bifidobacterium*, Bifidobacteriaceae, Ruminococcaceae, Verrucomicrobiaceae, and Christensenellaceae and decreases of *Roseburia*, *Faecalibacterium*, Lachnospiraceae ND3007 group, Prevotellaceae, *Blautia*, *Coprococcus*, and *Lachnospira* have been detected in patients with PD [17,90,91].
		Oral administration of berberine promotes l-DOPA production by *Enterococcus faecalis* and thus increases brain dopamine in PD model mice [93].	The metabolism of l-DOPA and dopamine is associated with the abundance of *Enterococcus faecalis* in patients with PD [92].
Autism	Glutamate, Dopamine, Serotonin, GABA, Acetylcholine, Histamine	Autism is associated with altered levels of glutamate, dopamine, GABA, acetylcholine, histamine, and serotonin in the brain of autism model mice/rat [99,100,101].	Autism is associated with the dysregulation of glutamate, dopamine, GABA, acetylcholine, histamine, and serotonin in the brain of patients with autism [99,100,101].
		The transplantation of feces from children with autism induces autism-like behaviors as well as altered tryptophan and serotonin metabolism in germ-free recipient mice [103]. Decreases in the serum levels of GABA and norepinephrine as well as increases in the serum level of serotonin and the abundance of *Lactobacillus* and *Collinsella* have been observed in an autism rat model transplanted with fecal samples from children with autism [104].	Increases of *Bacteroides*, *Prevotella*, *Lachnospiracea_incertae_sedis*, and *Megamonas* as well as decreases of *Clostridium XlVa*, *Eisenbergiella*, *Clostridium IV*, *Flavonifractor*, *Escherichia*/*Shigella*, *Haemophilus*, *Akkermansia*, and *Dialister* have been detected in patients with autism [102]. Gut microbial changes are associated with glutamate metabolism alteration in patients with autism [36].
		*Lactobacillus reuteri* can reverse social deficits in mouse models of autism [105].	
Schizophrenia	Dopamine	Mice transplanted with fecal microbiota from patients with schizophrenia exhibit impaired learning and memory abilities, reduced serum levels of tryptophan and serotonin, and increased levels of dopamine and serotonin in the prefrontal cortex and hippocampus, respectively [23].	Schizophrenia is characterized by the dysregulation of the dopamine system [59]. An increase of *Veillonella* and decreases of *Ruminococcus* and *Roseburia* have been detected in patients with schizophrenia [123]. Patients with schizophrenia exhibit increased abundance in facultative anaerobes in the gut and oral bacteria; altered metabolism of tryptophan; and altered synthesis pathways of glutamate, GABA, dopamine, and serotonin [110].
		*Streptococcus vestibularis* can induce neurotransmitter alterations in peripheral tissues and schizophrenia-like social behaviors in recipient mice [110].	
Anxiety	GABA, Serotonin, Glutamate, Dopamine	Anxiety is associated with the dysregulation of GABA, serotonin, and glutamate levels in the brain of anxiety model mice [113,124].	The dysregulation of serotonin, GABA, glutamate, and dopamine system in the brain is associated with the pathogenesis of anxiety disorders [64,111,112].
		*Lactobacillus rhamnosus* can increase GABA_B1b_ mRNA while reducing the GABA_Aα2_ mRNA level in the cortex, thereby ameliorating anxiety and depression-like behaviors in mice [75]. The anxiolytic-like biochemical characteristics observed in germ-free mice are associated with elevated hippocampal concentrations of serotonin and its main metabolite 5-HTP, an increased plasma concentration of its precursor tryptophan [70], and reduced expression of glutamate NMDA receptors and serotonin receptors in the cortex, hippocampus, or amygdala [73,74].	An increase of Burkholderiaceae, *Tyzzerella* 3, Betaproteobacteriales, *Hungatella*, *Escherichia/Shigella*, Enterobacteriaceae, Enterobacteriales, Bacteroidaceae, and Bacteroides and decreases of Prevotellaceae, *Prevotella* 9, *Dialister, Eubacterium_coprostanoligenes* group, *Subdoligranulum, Megamonas, Agathobacter*, Muribaculaceae, Ruminococcaceae_UCG-014, *Coprococcus* 1, Lachnospiraceae NK4A136 group, *Clostridium innocuum* group, *Buchnera*, Ruminococcaceae NK4A214 group, Tenericutes, Mollicutes_RF39_norank, Mollicutes, *Eubacterium xylanophilum* group, *Coprococcus* 3, *Eubacterium ruminantium* group have been detected in patients with anxiety [125].
Depression	GABA, Serotonin, Dopamine	An increase in Ruminococcaceae, and Porphyromonadaceae and decrease in Lactobacillaceae have been observed in a mouse model of depression, and the transplantation of its fecal microbiota induces depressive-like behaviors as well as altered endocannabinoid signaling in the hippocampus of germ-free recipient mice. Complement with *Lactobacillus plantarum* restores the behaviors by increasing fatty acid precursors of endocannabinoid ligands [116].	Depression is associated with the dysregulation of GABA, serotonin, and dopamine levels in patients with depression [114,115]. GABA is largely produced by *Bacteroides* in the gut microbiota. Correlation analysis of 16S rRNA sequencing and fMRI data shows that the relative abundance of *Bacteroides* in the feces of patients with depression is negatively correlated with brain symptoms of depression [19]. Subsequent transcriptomic analysis of human fecal samples corroborates that GABA is synthesized by these microbes [19].

### 3.5. Gut Microbiota as a Therapeutic Target for Neurological Disorders

There is accumulating evidence that the intestinal microbiome can directly or indirectly influence the nervous system through various mechanisms including blood delivery of microbe-derived products to the brain, through intestinal neuropod cells acting on the vagus nerve, through activation of intestinal endocrine cells to produce serotonin, and by affecting immune cells and inflammation. These findings support the microbiota–gut–brain axis hypothesis and have led to the discovery of microbe-derived drugs for the treatment of brain diseases [126]. Notably, microbial therapies based on GABA-producing intestinal bacteria for irritable bowel syndrome and insomnia are in development [126]. While microbial therapy holds immense promise for the treatment of brain disorders, major challenges include confirming the causality of such relationships and developing individualized treatments.

In addition, various medications including antidepressants, antipsychotics, and anticholinergics greatly affect microbial physiology and alter the intestinal microbiome structure [127,128,129]. Therefore, the development of drugs for neurological disorders must consider effects on the physiology of the gut microbiome, because microbial metabolism might positively or negatively affect drug efficacy. A recent study identified a multimicrobial interactive pathway based on the host’s dopamine metabolism whereby l-DOPA is first decarboxylated to dopamine by *Enterococcus faecalis* followed by dehydroxylation to *m*-tyramine by *Eggerthella lenta* [92]. Furthermore, single nucleotide polymorphisms in bacterial dopamine dehydroxylase genes can predict an individual’s ability to metabolize a given drug.

The recent development and application of technologies and methods such as abdominal windows, spatial transcriptomics, and circuit-tracing approaches have also promoted research on the relationship between the intestinal microbiome and enteric nervous system [130,131,132]. The enteric nervous system is the largest peripheral nervous system and is essential for gut motility and transducing intestinal sensory information to the brain. However, the enteric nervous system had remained enigmatic until the recent development of new means to monitor its activity in vivo. For instance, the invention of the abdominal window has enabled the simultaneous and direct observation and electrical recording of the enteric nervous system. Thus, by enabling the examination of the response of the enteric nervous system to drugs, neurotransmitters, or other stimuli, abdominal windows can help therapeutic development [131].

Thus, the gut microbiota is beginning to be appreciated as a source of potential therapeutic targets for neurological disorders, and, because of its critical role in the microbiota-gut-brain axis, offers great opportunities for the development of personalized medicine.

## 4. Perspectives

There is growing evidence that gut microbiota plays an important role in the pathogeneses of neurological diseases via the gut–brain axis. The metabolites, molecules, and endotoxins released by intestinal bacteria potentially affect the expression levels of neurotransmitters as well as their precursors and receptors in the central nervous system via the blood stream or vagus nerve pathways, thereby affecting brain function and cognitive behavior. However, the underlying molecular mechanisms are only beginning to be revealed.

Given the development of microbial therapies for brain disorders, future neurodrug design will critically depend on both the regulation of neurotransmitters and gut microbiota responsible for their biosynthesis. In addition, fecal microbiota transplantation of disease-associated bacterial taxa has potential therapeutic utility for some neurological disorders such as autism spectrum disorder and schizophrenia. Moreover, gut microbes may be useful for validating individuals’ metabolic responses to specific drugs, enabling accurate dosing and personalized therapy.
